# Meaningful Relationships in Community and Clinical Samples: Their Importance for Mental Health

**DOI:** 10.3389/fpsyg.2022.832520

**Published:** 2022-05-12

**Authors:** Victoria J. Block, Elisa Haller, Jeanette Villanueva, Andrea Meyer, Charles Benoy, Marc Walter, Undine E. Lang, Andrew T. Gloster

**Affiliations:** ^1^Department of Psychology, Clinical Psychology and Intervention Science, University of Basel, Basel, Switzerland; ^2^Psychiatric University Clinics (UPK), University of Basel, Basel, Switzerland; ^3^Division of Clinical Psychology and Epidemiology, Department of Psychology, University of Basel, Basel, Switzerland; ^4^Centre Hospitalier Neuro-Psychiatrique Luxembourg (CHNP), Ettelbruck, Luxembourg

**Keywords:** relationship quality, attributes, functions, wellbeing, community, psychiatric patients

## Abstract

Meaningful relationships are centrally important for human functioning. It remains unclear, however, which aspects of meaningful relationships impact wellbeing the most and whether these differ between psychiatric patients and members of the community. Information about relationship attributes and functions were collected in community members (*N* = 297) and psychiatric patients (*N* = 177). Relationship attributes and functions were examined for differences between groups (community vs. patients), their impact on wellbeing and symptoms, and the size of network (one vs. many relationships). Community members reported fewer relationships, higher frequency of contact and less desire for change when compared to the psychiatric patients. Nevertheless, both groups reported relatively high levels of fulfilled functions. Quality of the relationship and investment into the relationship was associated with both wellbeing and symptoms for both the community and the patient group. Almost all functions were associated with wellbeing and symptoms for the community group. However, for the patient group, only few functions (sexual partner, go-to person for compassion, go-to person when happy) were associated with wellbeing and no functions were associated with symptoms. Contrary to our hypotheses, the results show that psychiatric patients do not have a deficit in fulfilling relationships. Most people report a well-functioning network of meaningful, high-quality relationships. Patients benefit from meaningful, function-fulfilling relationships just as much as community members. Results are discussed with respect to how targeting relationships can be used clinically.

## Introduction

Humans are social animals (Tomasello, 2014) and therefore meaningful relationships are central to human’s existence (Gloster et al., 2021). Meaningful social relationships encompass all those close relationships that fulfill certain attributes and functions, and motivate someone to want to stay in touch with that person. Typically, meaningful relationships include individuals within the closest social network, such as spouses/partners, family members, or friends (Umberson and Karas Montez, 2010). Drawing upon a network of meaningful ties not just offers practical help, emotional support, safety, and ultimately serve survival (Holt-Lunstad et al., 2010), but also enables a life full of wellbeing, satisfaction and meaning. Meaningful relationships are characterized by a strong connectedness through reciprocal care and fulfillment of functions for each individual (Meiden et al., 2020). Indeed, various need fulfillments have been attributed to meaningful relationships, including social needs, such as belonging, being accepted, being valued as a person, or the social sharing of emotions (Rimé et al., 1992). Meaningful relationships also serve to fulfill personal basic needs such as the experience of autonomy, competence, and relatedness (Deci and Ryan, 2000), in turn predicting better relationship functioning and wellbeing (Patrick et al., 2007). Furthermore, meaningful relationships are often of high quality, which may also positively impact a person’s willingness to disclose personal issues and seek advice or help when needed (Robinson et al., 2008).

If meaningful relationships are of high quality, they can have a positive impact on our health and wellbeing (Kanter et al., 2018). High quality relationship can be defined as a relationship that fulfill functions such as providing social support (Green et al., 2002). Having high quality relationships has shown to be a protective factor for mental health, preventing from detrimental experiences such as stress and depression (Fuller-Iglesias et al., 2015; Newland, 2015). Several different categories of meaningful relationships have been shown to positively influence one’s life. Long-term romantic partners are a well-researched type of meaningful relationship and have shown to contribute to need fulfillment as well as act as a mood stabilizer (Kirchier, 1988). Another category that can act as a buffer for mental health struggles is close family members such as parents or siblings (Letourneau et al., 2013). Even meaningful relationships with friends (Parker et al., 2015) and co-workers (Wallace and Lemaire, 2007) have shown to be strong predictors of wellbeing. It is therefore not only social inclusion or the pure number of relationships that is important for wellbeing (Williams, 2007), but that a person can maintain satisfying, high quality meaningful relationships (Antonucci et al., 1997).

Even though humans can benefit from meaningful relationships (Clark and Lemay, 2010), the experienced protective effect may hinge on how the relationship characteristics are perceived by the individual in terms of quality, availability, satisfaction, and other attributes (Fletcher et al., 2000). When meaningful relationships are lacking or not fulfilling their function, it can have negative effects, such as increased stress (Randall and Bodenmann, 2017), which in turn increases the risk for developing mental health issues (Sheets and Craighead, 2014). For example, deficits perceived in relationships have shown to predict anxiety and depression in later life (Jacobson and Newman, 2016). Furthermore, continuous stressors, such as when a meaningful person suffers from anxiety or depression, increases the risk of transferring these symptoms, which is especially important for younger individuals (Lieb et al., 2002; Ginsburg et al., 2015). In such a case, a meaningful relationship might not be a positive factor for mental health and a person might not be mentioning these (former) relationships as a resource because of past estrangement (Tracy and Whittaker, 1990). When meaningful relationships are the source of stress or even hinder receiving appropriate support, the beneficial properties of meaningful relationships may simply become lost (Umberson and Karas Montez, 2010). The lack or impairment of meaningful social relationships has therefore been identified as a risk factor for poor mental health and increased mortality (Holt-Lunstad and Smith, 2012).

It is generally accepted that psychiatric patients’ relationships seem to be lacking (Brugha et al., 1993). Half of severely mentally ill patients have stated that they experience loneliness, which was highly associated with their poor wellbeing (Borge et al., 1999). Research on the social relationships of patients suffering from schizophrenia have found that half reported needing more meaningful interactions (Clinton et al., 1998). Additionally, for sufferers of chronic depression, aspects of social functioning can be severely impaired, making it difficult to uphold meaningful relationships (Kupferberg et al., 2016). When suffering from mental illness, adequately initiating, and keeping relationships can be challenging (Perese and Wolf, 2005), putting more pressure on existing relationships, especially if there is only one person available. Furthermore, depression can impair the perception of received social support (Ben-Zeev et al., 2009), curtailing the experience of need fulfillment in relationships. Regarding individuals suffering from mental illness, research suggests that lower social and emotional support as well as higher levels of loneliness are both associated with lower levels of wellbeing and mental health (Meltzer et al., 2013). However, making reciprocal meaningful connections where patients can give and received social support, has been found important for recovery of severe mental illness (Salehi et al., 2019), underlining the importance of meaningful relationships.

Patients struggling with mental illness often require increased care and support and prior research suggests that this is often provided by people classified as meaningful relationships such as a spouse or close family (Baronet, 1999). However, this can cause strain on those relationships and in extreme cases the burden of caregiving can cause burnout or mental illness to the meaningful person, especially if they are the only ones available and therefore are relied on heavily (Idstad et al., 2010). There is therefore the possibility that having only one relationship may be a future risk factor, as there is an increased probability of this important person no longer being available. Knowledge about the number of meaningful relationships would help a clinician to intervene and make building new or strengthening existing relationships a priority of treatment. So far, it is unknown which proportion of the general population have only one meaningful relationship and if the rates are similar for patients. To our knowledge, no detailed comparisons of clinical and non-clinical groups’ meaningful relationships exist. Previous research in clinical populations have often focused on social functioning and interpersonal problems in severe mental illness such as schizophrenia and major depressive disorder (Stevens et al., 2009; Kupferberg et al., 2016). However, differentiated characteristics of existing relationships of patients suffering from common mental disorders have not been documented. If psychopathology in general has a negative impact on meaningful relationships, then this is expected to be discernible in the data. We therefore want to compare the self-reported meaningful relationships of the general to the clinical population.

A subjectively fulfilling social life is traditionally defined as the number of active relationships (Cacioppo et al., 2002) or the discrepancy between experienced and desired levels of contact with another individual (Perlman and Peplau, 1981). Other methods to capture relationships are observer-based instruments. The Social Network maps, for example, gathers relevant data on relationships in an interview with the respondent and creates network map based on the reported connections (Tracy and Whittaker, 1990). The Interview Schedule for Social Interaction targets relationship needs but includes distal relationships as well (Henderson et al., 1980). However, these methods do not consider the functions of meaningful relationships, but rather focus on the number and official role of the people in a larger social network (Pollet et al., 2011) and their support function (Pescosolido and Wright, 2004).

As previous measures focused on quantitative and broader descriptions, the detailed characteristics of meaningful relationships are generally not well documented. To better understand the properties of a meaningful relationship for different groups, this paper aims to propose a differentiated way of describing the attributes and functions of meaningful relationships. The current study uses a comprehensive self-report measure that includes information about the functions and attributes of each meaningful relationship listed by the respondent. Attributes are defined as characteristics to describe a relationship (such as quality, frequency of meeting, desire for change in the relationship), and functions are defined as areas of need fulfillment in a relationship. In doing so, this study offers a way to identify more and less impactful facets of relationships and how they relate to mental health.

### The Present Study

The aim of this study is to describe the attributes of meaningful relationships and the functions fulfilled by the most important relationship in patients presenting for treatment as compared to participants in the community at large. Further, we wanted to determine whether and which attributes and functions of relationships are associated with wellbeing and symptoms, and whether these associations differ between the patient and the community sample. Finally, we wanted to examine whether these differ between individuals reporting only one meaningful relationship vs. many.

We hypothesized (1) that with respect to attributes, relative to the community sample, the patient sample would report worse relationship attributes (e.g., lower quality of relationships, less reciprocity in relationships) and consequently a stronger desire for changes in the reported relationships; and that ratings of functions (e.g., being a confidant, giving advice, etc.) for the most important person would be higher in the community sample than in the patient sample; we also expect the patient sample to report fewer relationships than the community sample; (2) for both the community and the patient sample, we expected significant associations between how participants judge their relationships (i.e., relationship attributes), how they rate to which extent their relationships fulfill needs (i.e., relationship functions) and their relation to reported wellbeing (positive association) and symptoms (negative association); and finally (3) that the attributes and functions reported by the group with only one meaningful relationship in their lives would be different compared to the attributes reported by the group with many relationships in their lives.

## Materials and Methods

### Sample

The overall sample for this study consists of *N* = 474 participants and was joined from two separate studies. The sample of study 1 is an online convenience sample from a non-clinical community population (*n* = 297). The sample of study 2 (*n* = 177) consists of the baseline data of psychiatric patients that were part of a multi-clinic, controlled effectiveness trial at a psychiatric hospital.^1^ Patients’ primary diagnoses were affective disorders (31.1%), anxiety and phobia disorders (38.4%) obsessive compulsive disorder (20.3%), post-traumatic stress disorder (2.3%), somatoform disorders (5.1%), impulse disorders (2.3%), and substance related disorders (0.6%). Two thirds of the patient sample (65.5%) received more than one diagnosis.

Overall, 62.0% of the sample are female with no differences in gender distribution between the samples. On average, participants were 32.6 years old (range = 18–80, *SD* = 11.73), with the patient sample being slightly older on average than the community sample. Patients were all residents of Switzerland (100%), while the community sample reported being residents of Switzerland (37.04%), Germany (55.56%), or living in other countries (7.41%) which constitutes a large difference in distribution. Of the sample, 38.7% had been in psychological care before (for sample 1 this refers to the past 12 months), with a large difference in distribution of psychological care use between the patient and the community sample. When reporting their living arrangements, 21.21% of participants said they lived alone, 42.42% said they were living with a partner, and 36.37% reported other living arrangements with no significant differences in distribution between samples. Table 1 shows detailed sample characteristics reported per subsample.

**TABLE 1:** Sample characteristics of community and clinical sample.

	Total *N* = 474 *M* (*SD*)	Community *n* = 297 *M* (*SD*)	Patients *n* = 177 *M* (*SD*)
Age	32.71 (11.71)	31.32 (11.79)	35.21 (11.17)*
**Sex**			
Females	292 (62.00%)	198 (66.67%)	94 (54.02%)
**Residency**			
Switzerland	287 (60.55%)	110 (37.04%)	177 (100%)***
Germany	165 (34.81%)	165 (55.56%)	0 (0%)***
Other country	22 (4.64%)	22 (7.41%)	0 (0%)***
**Living arrangement**			
Alone	118 (23.74%)	63 (21.21%)	55 (31.07%)
With partner	185 (37.22%)	126 (42.42%)	59 (33.33%)
Other arrangement	171 (34.41%)	108 (36.37%)	63 (35.60%)
Previous mental health care	226 (47.68%)	52 (17.51%)	174 (98.30%)***
MHC-SF	37.41 (14.76)	42 (12.68)	28.92 (14.16)***
BSI-18	15.75 (13.44)	11.43 (11.55)	23.71 (13.08)***

Oslo Social Support Scale (OSS), MHC-SF, Mental Health Continuum Short Form; BSI-18, Brief Inventory Checklist; Effect size indicators for differences of mean between the community and the patient group calculated as Hedge’s g. Nominal data was compared using Chi Square test and effect size was calculated using Cramer’s V for Residency and Living Arrangements and Phi (φ) for Previous Mental Health Care, MHC-SF, and BSI-18. *, small effect; ***, large effect.

### Procedure

#### Study 1

This was a cross-sectional online study on various psychological characteristics, including information about the participant’s meaningful relationships. Respondents were recruited between October 2017 and January 2018 via advertisements online and via social media and stemmed mostly from Germany and Switzerland, with a small portion recruited from other countries. Participants were at least 18 years old and sufficiently proficient in German. After consenting to the study, they filled out an online survey consisting of a series of questionnaires. At the end of the survey, they could partake in an optional raffle. In total, we excluded 113 participants from the sample who never completed the questionnaire.

#### Study 2

All participants from this study were in- and out-patients recruited from the Psychiatric University Clinics (UPK) in Basel, Switzerland between May 2016 and November 2019 as part of a larger clinical trial (Gloster et al., 2022). Inclusion criteria for this study were: at least 18 years old, sufficient proficiency in German, ability to participate in therapy, at least one mental disorder as determined by a clinical interview, and the provision of written informed consent. From 246 screened patients, 25 refused to participate, and 44 had to be excluded because they did not fulfill the inclusion criteria. Eligible and consenting patients underwent baseline assessment consisting of the Structured Clinical Interview for DSM-IV Axis I Disorders (SCID, Wittchen et al., 1997) and a questionnaire battery administered by trained post-graduate psychology students. Details on the study methods and design can be found elsewhere (Villanueva et al., 2019).

### Measures

#### Functional Assessment of Relationships

The Functional Assessment of Relationships (FAR; Gloster, n.d.), is a self-developed self-report instrument that was developed for use in a longitudinal clinical trial under the name SNQAS (Villanueva et al., 2019), as no satisfactory measurement for relationships attributes and functions was available. The FAR was developed to assess several dimensions of meaningful relationships from the participant’s perspective in three parts. It is based on the Valued Living Questionnaire (VLQ; Wilson et al., 2010), a questionnaire that covers different value dimensions and their importance, and on functional analysis principles known from behavioral therapy (Yoman, 2008). Using this method, the measure queries a variety of known functions to describe their presence and importance. In the first part, the respondent is prompted to list at least one and up to 15 people that have played a role in their life in the last 4 weeks and indicate what area of life they belong to (e.g., family, friends, co-workers, etc.). Specifically, it asks for any relationships that are meaningful to the respondent. For each listed person, the participants rate several items regarding relationship attributes. Attributes inquired include the importance of that person (e.g., how important that person is to the respondent, scaled from 1—not at all to 7—extremely important), the frequency (e.g., how often the respondent keeps in touch with this person, scaled from 1—daily to 4—less than once per week) and quality of contact (e.g., how the respondent would score the quality of contacts, scaled from 1—very bad to 7—very good), how available that person is (e.g., how easy this person is to contact and is available when needed, scaled from 1—never available to 5—always available), the reciprocity of the relationship (e.g., how balanced the respondent thinks the relationship is and who gives more than the other person, scaled from 1—the respondent only gives and does not receive to 5—the respondent only takes and does not give in the relationship), how actively the participant invests into the relationship (e.g., how much energy, time, and resources the respondent feels they invest in the relationship, scaled from 1—not at all to 7—very actively), and if there exists a desire for change (e.g., if the respondent thinks that the relationship should change in some way, scaled from 1—no change to 7—complete change). The attributes-items of the first part of the FAR had a Cronbach’s α = 0.62 after z-transformation necessary due to different scaling of items. By counting the amount reported meaningful persons per participant in the first part of the questionnaire, the variable “number of relationships” can be created. We have opted to add it to the attributes dataset and include this variable in the analyses. In the second part, respondents choose their most important relationship and answer items regarding the function of that person and how well this person can fulfill the respective needs. Functions chosen during the development of the questionnaire reflect different needs that a meaningful relationship can fulfill and that have been shown to be important in building family ties, partnerships, and friendships. Functions inquired included the role of being a confidant (e.g., a person one can confide in and talk about personal issues), sexual partner (e.g., a person one can be intimate with and/or is a romantic partner), a relationship where the participant could complain (e.g., talk about issues that are perceived as negative), gossip (e.g., talk about others), receive advice (e.g., the person can offer solutions and actionable ideas for problem-solving), get support (e.g., person can give emotional or material support), comfort (e.g., person can give respondent what they need to feel better), and contact when sad (e.g., person can deal well with sadness) or happy (e.g., positive feelings can be shared with this person) or to have fun (e.g., person is enjoyable company and good for activities such as parties). The functions-items of the second part of the FAR had high internal consistency with Cronbach’s α = 0.82. All items are scaled from 1—very bad to 7—very good with the option to mark 0—not applicable for this person. The third part of the instrument, which assesses changes in the attributes and functions over time, was not considered for the current study.

#### Mental Health Continuum

The Mental Health Continuum Short Form (MHC-SF; Franken et al., 2018) is a 14-items self-report scale measuring positive mental health. The scale consists of three subscales that relate to emotional, psychological, and social wellbeing. Participants report how often they have experienced a feeling in the past month on a 6-point Likert scale from 0 — never to 5 — every day. The measure has been found to be a reliable and valid instrument for general populations (Cronbach’s α = 0.89; Lamers et al., 2011) and for clinical populations (Cronbach’s α = 0.92; Franken et al., 2018). Across our samples, the measure showed to have high reliability as well (Cronbach’s α = 0.93). The results are interpreted by a sum score of all items with higher scores equate higher wellbeing.

#### Brief Symptom Inventory

The Brief Symptom Inventory (BSI; Derogatis, 1993) is a short 18-item questionnaire that measures symptom severity of somatic, depressive, and anxiety symptoms. The 18 items are a subsample of identical items from the full 53 item Brief Symptom Checklist (BSCL; Franke et al., 2015). The intensity of symptoms is rated on a 5-point Likert scale from 0 — not at all to 4 — a lot. It has been found to have good psychometric properties, such as high internal consistency (Cronbach’s α = 0.93) for the Global Severity Index (GSI) (Franke et al., 2017). For our samples, we calculated a high internal consistency score of Cronbach’s α = 0.93 for the measure overall. Results are interpreted by creating a sum score called GSI from all items. Higher scores signify higher severity of reported symptoms overall. Participants in the psychiatric patient sample completed the full BSCL; in this manuscript only the 18 items corresponding to the BSI were used for comparison with the community sample.

### Data Analysis

As the data from both samples were taken from other studies, we conducted a *post hoc* analysis statistical power calculation for regression analysis. Assuming a medium effect and an α of 0.05, the samples of *n* = 297 (community) and *n* = 177 (patients), respectively, are large enough for the assumed results to have a power of 67–94%, depending on the predictor, with most predictors having a power > 80 for significant results. For Hypotheses 1 and 3, we used independent two-sample *t*-tests to compare interval-scaled variables of the FAR between groups. When comparing multiple pairs of means, tests were corrected using the Benjamini and Hochberg adjustment (Benjamini and Hochberg, 1995) to correct for multiple testing. For Hypotheses 2, several linear models were employed for the community and patient group separately with symptoms (e.g., sum score of BSI) and wellbeing (e.g., sum score of the MHC-SF) as dependent variables and attributes and functions as predictors. For the calculation of multiple linear models, no correction was added. The linear models of both groups were compared using least square means to estimate and compare the slopes of the fitted models. For all analyses, data was checked for methodological prerequisites, such as normality of distribution. Transformation of data was performed when variables were too skewed and did not satisfy the assumption of normality before performing *t*-tests. Decision for transformation to normalize data was based on inspection of skewness and kurtosis and since moderate skew was found, data was transformed using square-root transformation (Manikandan, 2010). The optimal transformation was chosen based on an inspection of histograms and density plots. All analyses have been conducted in Jupyter Lab (Kluyver et al., 2016) Version 3.1.0 and used the programming language R (R Core Team, 2018).

## Results

### Hypothesis 1—Relationship Attributes and Functions of Patients Rated Lower Compared to Community

Overall, participants reported having roughly four people (*M* = 4.01, *SD* = 3.06) in their networks. Contrary to our hypothesis, the community sample reported fewer individuals in their network (*M* = 3.62, *SD* = 2.75) than the patient sample (*M* = 4.66, *SD* = 3.42), *t*(335.30) = 3.51, *p* = 0.002. This is a small to medium effect size of Hedges’ *g* = 0.34. The largest part of the reported relationships were family members (34.7%) and friends (30.8%). About 69.4% of the sample reported their partner (329 out of 474 participants). More details about the distribution of the relationship categories can be seen in Table 2.

**TABLE 2:** Distribution of reported relationship by category for the overall, community, and patients sample.

	Total *N* = 474	Community *n* = 297	Patients *n* = 177
			
Number of relationships	1,890	1,074	816
Only one relationship	115 (24.3%)	83 (30.0%)	32 (18.1%)
Same household	480 (25.4%)	305 (28.4%)	175 (21.4%)
**Relationship category**			
Partner	329 (17.4%)	227 (21.1%)	102 (12.5%)
Child	109 (5.8%)	66 (6.1%)	43 (5.3%)
Other family	655 (34.7%)	344 (32.0%)	311 (38.1%)
Friend	582 (30.8%)	322 (30.0%)	260 (31.9%)
Work colleague	83 (4.4%)	43 (4.0%)	40 (4.9%)
Fellow student	28 (1.5%)	21 (2.0%)	7 (0.9%)
Medical staff	27 (1.4%)	8 (0.7%)	19 (2.3%)
Other	66 (3.5%)	32 (3.0%)	34 (4.2%)

*For 11 reported relationships the category descriptor was missing.*

The average frequency of contact reported by participants was approximately 2–3 times per week (*M* = 2.03, *SD* = 0.83). In line with our hypothesis, the community sample reported higher frequency of contact (*M* = 1.90, *SD* = 0.80, equaling daily to 2–3 times per week), than the patient sample (*M* = 2.23, *SD* = 0.84, equaling contact one to two times per week), *t*(358.91) = 4.21, *p* < 0.001 (lower scores represent higher frequency of contact). The mean difference between groups translates to a small to medium-sized effect (Hedges’s *g* = 0.40).

The desire for change in relationships was reported with an average of 2.20 (*SD* = 1.28). In line with our hypothesis, the community sample reported less desire for change (*M* = 2.09, *SD* = 1.25) than the psychiatric patients (*M* = 2.37, *SD* = 1.31). The difference of means was significant, *t*(365.54) = 2.52, *p* = 0.03. The effect size was small with a Hedges’ *g* = 0.25. The two groups did not differ regarding the remaining attributes.

Regarding relationship functions, the community and patient groups did not differ, both rated the functions equally high (no mean value below 5 on a scale from 1 to 7). More details can be found in Table 3. A Chi Square test showed that there were no significant association between the frequency of rating a function and group, X^2^(48, *N* = 474) = 60, *p* = 0.115. More than 80% of participants in the community group and more than 90% from the patient group rated almost all functions as applicable to their reported most important person. Two functions were rated as applicable less often: Only half rated the function of “sexual partner” as applicable, and about 1 in 5 participants did not list the function “good time” as applicable. For a detailed description of attributes and functions in both samples, please refer to Table 3.

**TABLE 3:** Means and standard deviations of the FAR for the community and patient sample.

	Total *N* = 474 *M* (*SD*)	Community *n* = 297 *M* (*SD*)	Patients *n* = 177 *M* (*SD*)
**Attributes**			
Size	4.01 (3.06)	3.47 (2.75)	4.66(3.43)*
Quality	5.68 (1.32)	5.66 (1.45)	5.73 (1.06)
Importance	6.04 (1.17)	6.01 (1.30)	6.10 (0.89)
Frequency	2.03 (0.83)	1.90 (0.80)	2.24(0.83)**
Availability	3.93 (0.85)	3.96 (0.93)	3.88 (0.71)
Reciprocity	2.92 (0.49)	2.90 (0.56)	2.96 (0.36)
Investment	5.19 (1.48)	5.22 (1.54)	5.13 (1.38)
Desire for change	2.18 (1.27)	2.09 (1.25)	2.33(1.29)*
**Functions**			
Confidant (*n* = 439)	6.44 (0.96)	6.35 (1.08)	6.59(0.71)**
Sexual partner (*n* = 231)	5.62 (1.72)	5.81 (2.57)	5.24(1.94)*
Complaint (*n* = 436)	5.89 (1.27)	5.97 (1.23)	5.76 (1.31)
Gossip (*n* = 427)	5.73 (1.41)	5.76 (1.36)	5.67 (1.47)
Advice (*n* = 440)	6.02 (1.21)	6.07 (1.17)	5.95 (1.27)
Support (*n* = 441)	6.28 (1.05)	6.28 (1.11)	6.27 (0.94)
Comfort (*n* = 441)	5.95 (1.22)	5.97 (1.30)	5.9 (1.08)
Go to when sad (*n* = 425)	5.70 (1.45)	5.80 (1.40)	5.55 (1.5)
Go to when happy (*n* = 429)	6.11 (1.17)	6.19 (1.10)	5.99 (1.26)
Go to for fun (*n* = 386)	5.39 (1.46)	5.36 (148)	5.44 (1.44)

*Size is the number of people reported in the FAR; quality was rated from 1 (very bad) to 7 (very good); importance was rated from 1 (not at all important to) 7 (extremely important); Frequency was rated from 1 (daily contact) to 4 (less than once weekly); Availability was rated from 1 (never available) to 4 (always available); Reciprocity was rated from 1 (person only takes) to 5 (person only gives); Investment was rated from 1 (no active investment at all) to 7 (very active investment); Desire for Change was rated from 1 (no change) to 7 (complete change).*

The quality of functions was rated from 1 (very bad) to 7 (very good). Respondents could select “does not apply to this person,” hence the changing n for each item. Significance indicators for differences of mean between the community and the patient group: *p < 0.01, **p < 0.001.

### Hypothesis 2—Relationship Attributes and Functions Are Associated With Wellbeing and Symptoms

Overall, the community group reported significantly higher levels of wellbeing (*M* = 42.44, *SD* = 12.68) than the patient group (*M* = 28.92, *SD* = 14.16), *t*(335.96) = −10.43, *p* < 0.001. At the same time, the community group reported lower overall depressive, anxiety, and somatic symptoms (*M* = 11.43, *SD* = 11.55) compared to the patient group (*M* = 23.71, *SD* = 13.08), *t*(295.41) = 9.99, *p* < 0.001. For details on the subscales for MHC-SF and BSI, please refer to Table 1.

For both the community sample and the patient sample, some attributes were associated with wellbeing and symptoms. Relationship quality and level of investment into the relationship were both positively associated for the community and the patient group while a higher desire for change was associated with lower wellbeing for both groups. More frequent contact (lower frequency of contact scores) was associated with higher wellbeing for community members exclusively while the importance of the relationships was positively associated for the patient group only. The number of relationships, availability for contact, and the experienced reciprocity of the relationships did not siginificantly predict wellbeing.

Most relationship attributes were also associated with symptoms, albeit not all and not for both groups. Relationship quality, availability for contact, and relationship investment were all negatively association with symptom severity for both groups. For the community group but not for patients, relationship importance and reciprocity were also negatively associated with symptoms. On the other hand, a desire for change was positively associated with symptom severity for the patients but not for the community group. The number of relationships and the frequency of contact was not associated with symptom severity for either group. When comparing the associations of both groups with each other (beta coefficients) none of the group’s analyses differed.

Regarding functions, almost all relationship functions were significantly associated with wellbeing for the community group. Specifically, all functions except “I can gossip with this person” were positively associated with wellbeing for the community group. For the patient group, however, only the function of “I go to this person when I am happy,” “sexual partner,” and “this person gives me compassion” were significantly associated with wellbeing, with the latter two being only marginally significant. All other functions were not associated with wellbeing for the patient group.

Furthermore, functions were almost all negatively associated with symptom severity for the community group with the exceptions being “I can gossip with this person” and “I go to this person when I’m sad.” However, none of the functions were significantly associated with symptom severity for the patient group. While the groups’ regression analyses’ slopes did not differ when associating relationship functions with wellbeing, the beta coefficients of the slopes did differ between the groups when comparing the association between “Trusted,” “sexual partner,” and “compassion.” Detailed results and between-group analyses can be found in Table 4 (Attributes) and Table 5 (Functions). For visualized regression slopes for each attribute and function with wellbeing and symptoms separated by group, please refer to Supplementary Figures A–D.

**TABLE 4:** Linear regressions for relationship attributes with wellbeing or symptoms for the community and the patient sample.

		Community (*n* = 297)				Patients (*n* = 177)					
		Wellbeing								Comparison	
Predictor	Attributes	Intercept	β	*R* ^2^	*p*	Intercept	β	*R* ^2^	*p*	Slope Δ	*p*
	Number of relationships	43.045	−0.167	0.001	0.533	28.113	0.173	0.002	0.581	−0.34	0.402
	Quality	34.676	1.362	0.024	**0.008**	12.533	2.867	0.045	**0.005**	−1.51	0.166
	Importance	37.394	0.830	0.007	0.150	9.041	3.265	0.042	**0.006**	−2.43	0.056
	Frequency of contact	47.551	−2.721	0.029	**0.004**	34.478	−2.483	0.021	0.052	−0.24	0.877
	Availability for contact	36.313	1.532	0.013	0.057	18.948	2.569	0.017	0.089	−1.04	0.528
	Reciprocity	46.745	−1.507	0.004	0.265	33.085	−1.410	0.001	0.640	−0.10	0.976
	Investment	33.832	1.637	0.039	**<0.001**	16.878	2.362	0.053	**0.002**	−0.73	0.042
	Desire for change	49.066	−3.205	0.098	**<0.001**	32.913	−1.681	0.025	**0.038**	−1.52	0.112

		**Symptoms**								**Comparison**	
**Predictor**	**Attributes**	**Intercept**	β	***R* ^2^**	***p***	**Intercept**	β	***R* ^2^**	***p***	**Slope **Δ****	***p***

	Number of relationships	10.971	0.127	< 0.001	0.603	21.411	0.491	0.017	0.104	−0.36	0.336
	Quality	31.162	−3.477	0.190	**<0.001**	45.505	−3.816	0.095	**<0.001**	0.34	0.723
	Importance	25.876	−2.395	0.073	**<0.001**	35.370	−1.923	0.017	0.095	−0.47	0.687
	Frequency of contact	13.018	−0.806	0.003	0.344	21.526	0.967	0.004	0.441	−1.77	0.226
	Availability for contact	28.243	−4.233	0.117	**<0.001**	36.974	−3.427	0.034	**0.019**	−0.81	0.592
	Reciprocity	27.487	−5.522	0.071	**<0.001**	31.969	−2.801	0.006	0.333	−2.72	0.350
	Investment	25.332	−2.651	0.125	**<0.001**	31.683	−1.570	0.029	**0.031**	−1.08	0.170
	Desire for change	9.616	0.896	0.009	0.101	19.057	1.964	0.040	**0.011**	−1.07	0.243

*Slope Δ stands for the difference in slopes between the patient and the community group and p shows if the slopes differ significantly between the groups.*

*p-values in bold font highlight statistically significant associations of an attribute with the well-being or the symptoms outcome respectively.*

**TABLE 5:** Linear regressions for relationship functions with wellbeing or symptoms for the community and the patient sample.

		Community (*n* = 297)				Patients (*n* = 177)					
		Wellbeing								Comparison	
Predictor	Attributes	Intercept	β	*R* ^2^	*p*	Intercept	β	*R* ^2^	*p*	Slope Δ	*p*
	Trusted	23.757	2.911	0.065	**<0.001**	18.040	1.738	0.008	0.259	−1.12	0.169
	Sexual partner	34.103	1.627	0.045	**0.008**	19.490	1.815	0.054	**0.047**	0.27	0.531
	Complain	32.417	1.655	0.028	**0.007**	26.274	0.435	0.002	0.602	1.22	0.222
	Gossip	36.215	1.088	0.015	0.051	23.438	0.965	0.010	0.198	0.12	0.892
	Advice	29.110	2.160	0.041	**<0.001**	19.618	1.556	0.020	0.069	0.60	0.562
	Support	22.889	0.062	0.075	**<0.001**	17.369	1.860	0.015	0.107	1.20	0.343
	Compassion	26.642	2.584	0.071	**<0.001**	17.385	1.973	0.023	**0.050**	0.61	0.580
	Sad	30.462	2.050	0.052	**<0.001**	22.250	1.231	0.017	0.097	0.82	0.360
	Happy	28.167	2.271	0.039	**0.001**	12.837	2.774	0.061	**0.001**	−0.50	0.642
	Good time	33.967	1.553	0.034	**0.004**	23.831	1.023	0.010	0.218	0.53	0.575

		**Symptoms**								**Comparison**	
**Predictor**	**Attributes**	**Intercept**	β	***R* ^2^**	***p***	**Intercept**	β	***R* ^2^**	***p***	**Slope Δ**	***p***

	Trusted	25.543	−2.343	0.069	**<0.001**	25.711	0.322	<0.001	0.827	−2.35	**0.001**
	Sexual Partner	18.645	−1.596	0.069	**<0.001**	24.790	−0.118	< 0.001	0.886	−1.06	**0.009**
	Complain	17.904	−1.204	0.023	**0.013**	23.411	0.098	< 0.001	0.905	−1.30	0.141
	Gossip	14.056	−0.627	0.008	0.151	24.542	−0.074	< 0.001	0.917	−0.55	0.478
	Advice	18.848	−1.358	0.027	**0.007**	24.832	−0.163	< 0.001	0.840	−1.19	0.178
	Support	18.272	−1.233	0.021	**0.018**	22.978	0.106	< 0.001	0.929	−1.34	0.247
	Compassion	18.599	−1.329	0.031	**0.004**	16.217	1.260	0.011	0.189	−2.59	**0.007**
	Sad	14.719	−0.728	0.011	0.093	22.135	0.309	0.001	0.662	−1.04	0.181
	Happy	20.618	−1.623	0.034	**0.003**	23.595	−0.028	< 0.001	0.973	−1.60	0.090
	Good Time	15.218	−0.868	0.017	**0.046**	27.006	−0.732	0.007	0.338	−0.14	0.867

*Slope Δ stands for the difference in slopes between the patient and the community group and p shows if the slopes differ significantly between the groups.*

*p-values in bold font highlight statistically significant associations of an attribute with the well-being or the symptoms outcome respectively.*

### Hypothesis 3—Relationship Attributes, but Not Functions, Differ Between Participants With Only One vs. Many Relationships

For the last analysis, the total sample was split in two groups: those who report only one (*n* = 115) vs. those reporting multiple meaningful relationships (*n* = 369). Overall, a larger proportion of participants from the community sample reported only one relationship compared to the patient sample [30.0% vs. 18.1%, *t*(405.1) = −2.43, *p* = 0.016]. Participants with only one meaningful relationship differed from participants with multiple meaningful relationships in four out of seven attributes: They reported more frequent contact (*M* = 1.53, *SD* = 0.92) than the group with more relationships (*M* = 2.19, *SD* = 0.73), *t*(160.84) = 6.97, *p* < 0.001 (as lower values equal more frequent contact). This results in a large effect size with Hedges’ *g* = 0.85. Furthermore, participants with only one meaningful relationship reported higher quality (*M* = 5.97, *SD* = 1.59) and higher importance (*M* = 6.43, *SD* = 1.30) of that relationship, compared to the average of the group with several reported relationships (quality: *M* = 5.59, *SD* = 1.19, importance: *M* = 5.91, *SD* = 1.09). These attributes showed a small to medium (Hedge’s *g* = 0.30) and a medium (Hedge’s *g* = 0.45) effect size, respectively. Lastly, the group with only one relationship reported a higher levels of investment into this relationship (*M* = 5.68, *SD* = 1.67) compared to the group with several relationships (*M* = 5.02, *SD* = 1.38), which results in a medium effect size (Hedge’s *g* = 0.45). The groups did not differ in the other relationship attributes.

The groups also did not differ in any of the functions assigned to their most important person. Chi Square test showed that there were no significant associations between the frequency of rating a relationship function and group, X^2^(54, *N* = 474) = 60, *p* = 0.267. The functions that were rated fewest by both groups, “sexual partner” and “good time,” were rated equally often in the group with one vs. multiple relationships. More details can be found in Table 6.

**TABLE 6:** Means and standard deviations of the FAR for one relationship vs. many relationships.

	Several relationships *n* = 359 *M* (*SD*)	Only one relationship *n* = 115 *M* (*SD*)
**Attributes**		
Size	4.97 (2.92)	1.00 (0.00)
Quality	5.59 (1.19)	5.97 (1.59)*
Importance	5.91 (1.09)	6.43 (1.30)**
Frequency	2.19 (0.73)	1.53 (0.92)**
Availability	3.88 (0.78)	4.08 (1.03)
Reciprocity	2.93 (0.45)	2.89 (0.60)
Investment	5.02 (1.38)	5.68 (1.67)**
Desire for change	2.21 (1.17)	2.15 (1.56)
**Functions**		
Confidant (*n* = 439)	6.47 (0.89)	6.34 (1.17)
Sexual partner (*n* = 231)	5.62 (1.73)	5.67 (1.67)
Complaint (*n* = 436)	5.86 (1.28)	6.00 (1.21)
Gossip (*n* = 427)	5.76 (1.43)	5.63 (1.32)
Advice (*n* = 440)	6.02 (1.22)	6.01 (1.20)
Support (*n* = 441)	6.29 (1.00)	6.22 (1.21)
Comfort (*n* = 441)	5.92 (1.22)	6.05 (1.23)
Go to when sad (*n* = 425)	5.66 (1.48)	5.83 (1.34)
Go to when happy (*n* = 429)	6.12 (1.19)	6.07 (1.11)
Go to for fun (*n* = 386)	5.41 (1.48)	5.34 (1.41)

*Size is the number of people reported in the FAR; quality was rated from 1 (very bad) to 7 (very good); importance was rated from 1 (not at all important to) 7 (extremely important); Frequency was rated from 1 (daily contact) to 4 (less than once weekly); Availability was rated from 1 (never available) to 4 (always available); Reciprocity was rated from 1 (person only takes) to 5 (person only gives); Investment was rated from 1 (no active investment at all) to 7 (very active investment); Desire for Change was rated from 1 (no change) to 7 (complete change).*

The quality of functions was rated from 1 (very bad) to 7 (very good). Respondents could select “does not apply to this person,” hence the changing n for each item. Significance indicators for differences of mean between the community and the patient group: *p < 0.01, **p < 0.001.

## Discussion

This study examined the importance of meaningful relationships of individuals in the community and in psychiatric treatment. Across both groups, the results suggest that people are embedded in a small but high-quality network of meaningful relationships. Importantly, these relationships affect wellbeing for both groups. In contrast, *how* people interact with the most important person in their lives was not related to symptoms for patients but it was for the community sample. Patients reported more meaningful relationships, a lower frequency of contact, and more desire for change of the *status quo* of the relationship than non-patients. The group of participants reporting only one important relationship attributes a higher level of importance to their relationship, invests more into the relationship, reports higher perceived quality and sees their meaningful person more often than the group of participants reporting several relationships. This study clearly showed that people view their relationships as a source of need fulfillment and support—and this is equally true for patients and community members.

### Hypothesis 1—Patients and Community Members Rate Their Relationships Equally High

Our first hypothesis, namely that patients report lower relationship attributes measures relative to the community, was partially confirmed. The samples differed on three measures: number of reported relationships, the frequency of contact, and the desire for change in the relationship. The overall sample reported having on average 4.01 meaningful relationships, which is a bit lower than the 6.75 meaningful relationships reported in a study that asked participants to list all people they know and want to stay in contact with (Pollet et al., 2011). However, patients revealed slightly larger networks of meaningful relationships (4.66) compared to community members (3.47). This contradicts an earlier study where people suffering from mental illness reported 2.5 smaller social networks (Koenders et al., 2017). Furthermore, patients in a previous study reported four relationships when listing social support (Pescosolido and Wright, 2004), which is similar to our results. Our findings suggest that patients may be able to rely on the same or more relationships compared to community members and the deficit might not be the amount of relationships that are experienced as meaningful. Furthermore, patients have described their relationships as a core resource in the recovery process (Schön et al., 2009), which might encourage them to focus on their social resources more. This is in line with the finding that meaningful relationships may influence how a person experiences their quality of life and how included they feel socially (Herbert, 2018). Consistent with this position, we found that compared to community members, patients reported a greater desire for change in their meaningful relationships. This may be partially explained by the fact that patients do not feel they have the capacity to fully invest in a reciprocal relationship (Salehi et al., 2019) or they expect the other person to drive the change. However, both the patient and non-patient groups exhibited high ratings of their relationships’ quality contrasting results from previous studies that found a lack of high-quality relationships and increased social isolation in people suffering from mental health issues (Kawachi and Berkman, 2001; Perese and Wolf, 2005).

Individuals in both patient and community groups reported similar levels of function fulfillment by their most significant person, with only few differences between the groups. Firstly, the role of a confidant was better fulfilled by the most important person of patients than of community members. Secondly, the subset of patients in romantic relationships were less satisfied with their sexual partners compared to the community sample, which is consistent with prior reports in clinical populations (McCann, 2003). We expected a lack of fulfilled functions in the clinical group, considering that psychosocial difficulties often are partly a reason for treatment (Perese and Wolf, 2005). However, the lack of differences suggests that both non-clinical and clinical groups report relationships that have the capacity to fulfill needs.

### Hypothesis 2—Relationship Attributes Are Associated With Wellbeing and Symptoms

The results showed that only half of the relationship attributes were associated with wellbeing and symptoms for both the community and the patient group. This means, that some attributes such as the number of meaningful relationships, the availability for contact, and the reciprocity did not significantly change with wellbeing. This partly contradicts a previous study that has found that both the frequency and the quality of social interactions in relationships matter for wellbeing (Sun et al., 2020). For both groups, however, the quality of and investment into the relationship was positively associated with wellbeing and with symptoms, supporting the hypothesis that individuals, irrespective of mental health status, value high quality interactions that motivate them to invest into a relationship. One way to see this is that a high quality rating may stand in for an overall satisfaction with the interactions experienced with this person (Fiorillo and Sabatini, 2011), even if they may be less frequent. Furthermore, the association of the desire for change in the relationship with wellbeing (positive) for both groups and with symptoms (negative) for patients is in line with our expectations. When wellbeing is low or symptoms are more severe, a common reaction would be to search for causes in one’s direct surrounding, often creating social conflicts (Darbonne et al., 2013). The results could therefore be an expression of a need to improve meaningful relationships, in order to feel better. This interpretation is supported by prior research where higher need fulfillment was associated with higher relationship satisfaction and less conflict (Patrick et al., 2007). Having people in one’s life that are willing to give support by being available when needed is negatively connected to symptom severity for both groups. This could mean that alleviating symptoms could be aided by meaningful relationships, such as close friends, that offer support and connection (Knickmeyer et al., 2002).

Overall, better fulfillment of functions was indicative of higher wellbeing and lower symptoms for the community sample. However, for patients, only few functions were related to wellbeing and not related to symptoms at all. It might be that the negative experience of suffering with a mental illness could decrease the sensitivity to need fulfillment in meaningful relationships. Another explanation could be that the patients experience a decreased interest in their meaningful relationships This has been observed in patients suffering from depressive disorders and can lead to relationship difficulties (Kupferberg et al., 2016). One function that was negatively related to wellbeing for patients was the function of a sexual partner. Since sexual functioning has been linked to wellbeing and mental health (Mernone et al., 2019), it is reasonable to assume that this function may be suffering from other factors related to the clinical group’s mental disorders. The strongest association with wellbeing for patients was the function of “I go to this person when I am happy.” Patients suffering from mental illness often experience a lot of negative emotions and difficult symptoms. Even brief emotional relief can be hard to find and one way this can be achieved is by experiencing connectedness in meaningful relationships (Howell et al., 2011).

Overall, relationship functioning appeared to have a distinctive influence on wellbeing and symptoms, however, only in the community sample. One can conclude that even though the non-clinical and clinical group alike are able to foster a meaningful relationship that fulfills key functions, patients did not benefit much from a positive association of need fulfillment with wellbeing. Furthermore, the lack of association of the patient’s symptoms with their funtion ratings may be explained by their higher symptom levels and, respectively, their experience of suffering regardless of fulfilled needs.

### Hypothesis 3—Reporting Only One Relationship Is Associated With Higher Attribute Ratings

Overall, one in four participants reported only one meaningful relationship. The proportion was higher in the community sample (30.0%) than in the patient sample (18.1%). Participants with only one meaningful relationship reported overall a higher quality, higher importance, and a higher personal investment into the relationship compared to those who reported multiple meaningful relationships. Previous research has documented that a single meaningful relationship can fulfill all needs of a person (Cohen and Wills, 1985), but others have found that few relationships are linked to higher symptoms (Barnett and Gotlib, 1988). Our data suggests that having only one relationship is not automatically detrimental as long as it is meaningful and serves need fulfillment (Patrick et al., 2007). It even suggests that one meaningful relationship can become the sole focus of one person because there is higher investment and thus can turn out very satisfying. Investing in this relationship is important, though the lack of other meaningful relationships may put the person at risk if this relationship were to disappear. Furthermore, people who report one meaningful relationship seem to rely fully and only on that relationship. This might put pressure or strain on the relationship, possibly causing long term negative effects for both the participant and their most important person (Perlick et al., 2016). This finding may be important for the treatment of patients who report having only one meaningful relationship. Carers of patients with mental illness often report psychological distress and reduced wellbeing but they also feel responsible for their wellbeing (Möller-Leimkühler and Wiesheu, 2012). Would patients with only one meaningful relationship be encouraged to build other relationships that can take over some of the functions, that strain would be distributed across several people and lift the burden off the one person. Another effect could be that patients would therefore be able to live a more balanced relationship with this person and maybe able to transform the relationship into the direction they would like, seeing that patients in our data wanted their meaningful relationships to change.

Relationship functions did not differ between individuals with one and those with many relationships. Both groups reported equal need fulfillment. This was contrary to our hypothesis as we expected that having only one relationship would put more negative pressure on this relationship to cover different relational needs (Žvelc et al., 2020). Furthermore, contrary to previous findings that people with few relationships can experience self-inflicted social isolation (Topor et al., 2006), we found that on average also those with only one relationship have their needs for social support met. On the other hand, it might be that the motivation to renew social connections to evade loneliness (Cacioppo et al., 2011) could be a driver behind these high functional ratings, especially for the group with only one relationship.

### Limitations

This study has several limitations. First, all relationships were rated highly. It could therefore be that participants of both samples underreported problematic relationships perhaps due to their perceived negative influence in their lives. Studies have found that people do report problematic or ambivalent relationships when specifically asked for it (Fingerman et al., 2004). However, participants in our studies only reported overall positively rated relationships. It is possible that problematic relationships were not considered meaningful and therefore not mentioned. Second, previous research suggests that subjective reporting on meaningful relationships, as implemented in this study, might generate inaccurate information (Tracy et al., 1990) due to recall bias and memory-experience gap (Rinner et al., 2019). However, studies have found that the recounts of relationships by study participants is very accurate and can be taken at face value (Wright and Pescosolido, 2002). Third, the samples differ regarding a few sample characteristics and have been collected in very different settings (online vs. self-administration with study personnel present). However, except for psychiatric care use and psychological variables, groups only differed regarding their age and country of residence. The difference in mean age between the groups was small and therefore retaining the whole sample was given priority over matching the samples for age. Concerning data collection it can be argued that due to the similarity between German and Swiss culture (Kopper, 1993), the differences in residency were not expected to have a large impact on the results. On the other hand, the method of data collection may have resulted in patients reporting on their meaningful relationships more positively due to socially desirable responses, perhaps overestimating their responses. However, there is evidence that this effect is small compared to self-administration (Heerwegh, 2009) and patients did fill out the questionnaires themselves mitigating this concern further even when the presence of study personnel could have cause a slight distortion. Lastly, the study design required respondents to fill in at least one person, which resulted in no respondents reporting zero meaningful relationships. Being without meaningful relationships could be worse than having at least one, as this has been associated with significantly lower wellbeing (Chappell and Badger, 1989). This concern was mitigated by the fact that participants entering only one person also reported high attribute and function-fulfillment throughout, regardless of clinical status.

## Conclusion

This study described the meaningful relationships of community members and psychiatric patients. It found that, irrespective of mental health status, participants report having relationships that are fulfilling and of good quality. Patients reported more relationships on average, showing good social support systems. Although a quarter of the respondents only reported one relationship, they did not experience this relationship as less need fulfilling than respondents who reported a larger network of meaningful relationships. Some relationship attributes were associated with wellbeing and symptoms both for the community and patient sample. Most importantly, need fulfillment was related to wellbeing and symptoms for the community sample, but not for the patients sample. While the community sample may profit from a focus on increasing need fulfillment, patient’s wellbeing and symptoms were largely disconnected from relationship attributes and functions. Patients might therefore have to take another approach such as actively working on their relationship attributes, voicing their desire for change, and invest more into their relationships.

### Future Research

Future research should compare the associations between relationship characteristics and well-being in patient groups and the general population in different cultural settings. This would allow to eventually compare samples across countries and possibly connect it to culturally relevant factors. Furthermore, malleable targets such as the investment into meaningful relationships, as well as the frequency of contact should be investigated to derive interventions that improve wellbeing and alleviate symptoms through social connections. Furthermore, investigating important relational functions in detail could help understand how these needs are met for both community members and people suffering from mental health issues. Understanding social needs is crucial, as they affect wellbeing, even in situations where basic needs stay unmet (Tay and Diener, 2011). Lastly, the results are associative in nature and provoke questions regarding the directionality of the observed relationships. Future research could investigate under which circumstances the influence of fulfilled relationship functions on wellbeing and symptoms wanes and how this could be used as a predictor of intervention outcomes.

### Implications for Clinical Work

This study clearly showed that people view their relationships as a source of need fulfillment and support. This is equally true for patients and community members. Due to the implication of the absence or impaired functionality of meaningful relationships on mental health and wellbeing (Lincoln, 2000), assessing and describing meaningful relationships could be an important part of risk assessment when treating a new patient. The finding that function fulfillment was not associated with wellbeing and symptoms for patients but for the community group could mean that this association may be an indicator for a person’s social functioning. Conversely, when having needs fulfilled stops being associated with wellbeing and experienced symptoms, this might be interpreted as a warning sign of deteriorating mental health. It might be important in clinical work to first focus on the attributes of meaningful relationships to reenable impactful function fulfillment in relationships. Understanding our meaningful relationships and how they can support us is crucial for the development of tangible interventions which aim at improving wellbeing for patients and for society.

## Data Availability Statement

The raw data supporting the conclusions of this article will be made available by the authors, without undue reservation, to any qualified researcher.

## Ethics Statement

The studies involving human participants were reviewed and approved by the Ethikkommission Nordwest- und Zentralschweiz (EKNZ, Project 165/13). The patients/participants provided their written informed consent to participate in this study.

## Author Contributions

AG and VB contributed to the conception and design of the study. VB, JV, and CB organized the database. VB performed the statistical analysis and wrote the first draft of the manuscript. AG and EH wrote sections of the manuscript. All authors contributed to manuscript revision, read, and approved the submitted version.

## Conflict of Interest

The authors declare that the research was conducted in the absence of any commercial or financial relationships that could be construed as a potential conflict of interest.

## Publisher’s Note

All claims expressed in this article are solely those of the authors and do not necessarily represent those of their affiliated organizations, or those of the publisher, the editors and the reviewers. Any product that may be evaluated in this article, or claim that may be made by its manufacturer, is not guaranteed or endorsed by the publisher.

## Footnotes

1 The study was registered with the ISRCTN: ISRCTN11209732 and approved by the Ethics Committee of northwestern and central Switzerland (Ethikkommission Nordwest- und Zentralschweiz; EKNZ): Project 165/13.

## Supplementary Material

Supplementary Figure A Visualization of the association of the relationship attribute ratings and wellbeing for both the community and the patient group.

Supplementary Figure B Visualization of the association of the relationship attribute ratings and symptoms for both the community and the patient group.

Supplementary Figure C Visualization of the association of the relationship function ratings and wellbeing for both the community and the patient group.

Supplementary Figure D Visualization of the association of the relationship function ratings and symptoms for both the community and the patient group.
