# Supplementary figures and images for: Meaningful Relationships in Community and Clinical Samples: Their Importance for Mental Health
Source: Front Psychol. 2022 May 12;13:832520. doi: 10.3389/fpsyg.2022.832520 (PMC9133738; doi:10.3389/fpsyg.2022.832520)

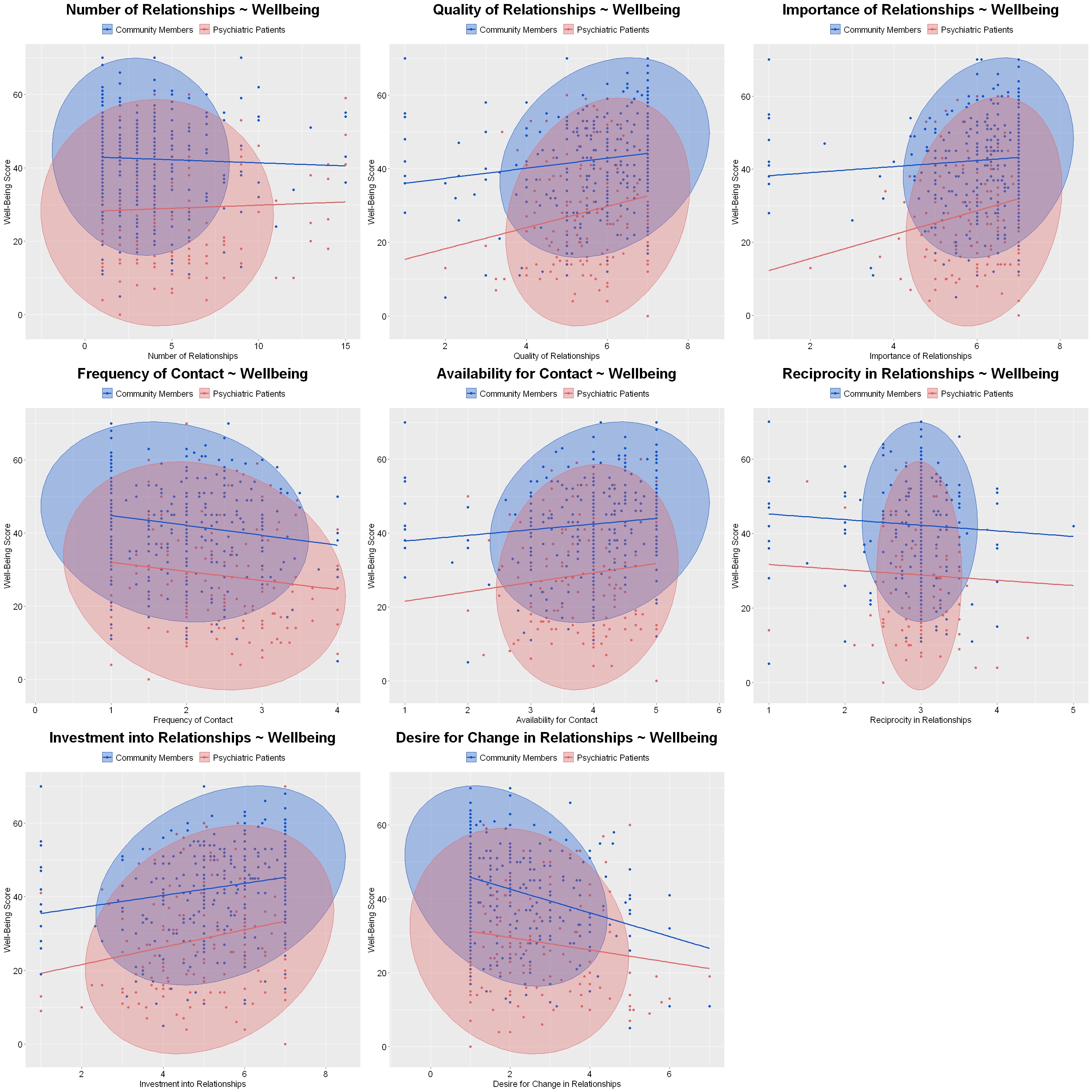

Supplement: Supplementary Figure A — Visualization of the association of the relationship attribute ratings and wellbeing for both the community and the patient group. [file Image_1.JPEG]

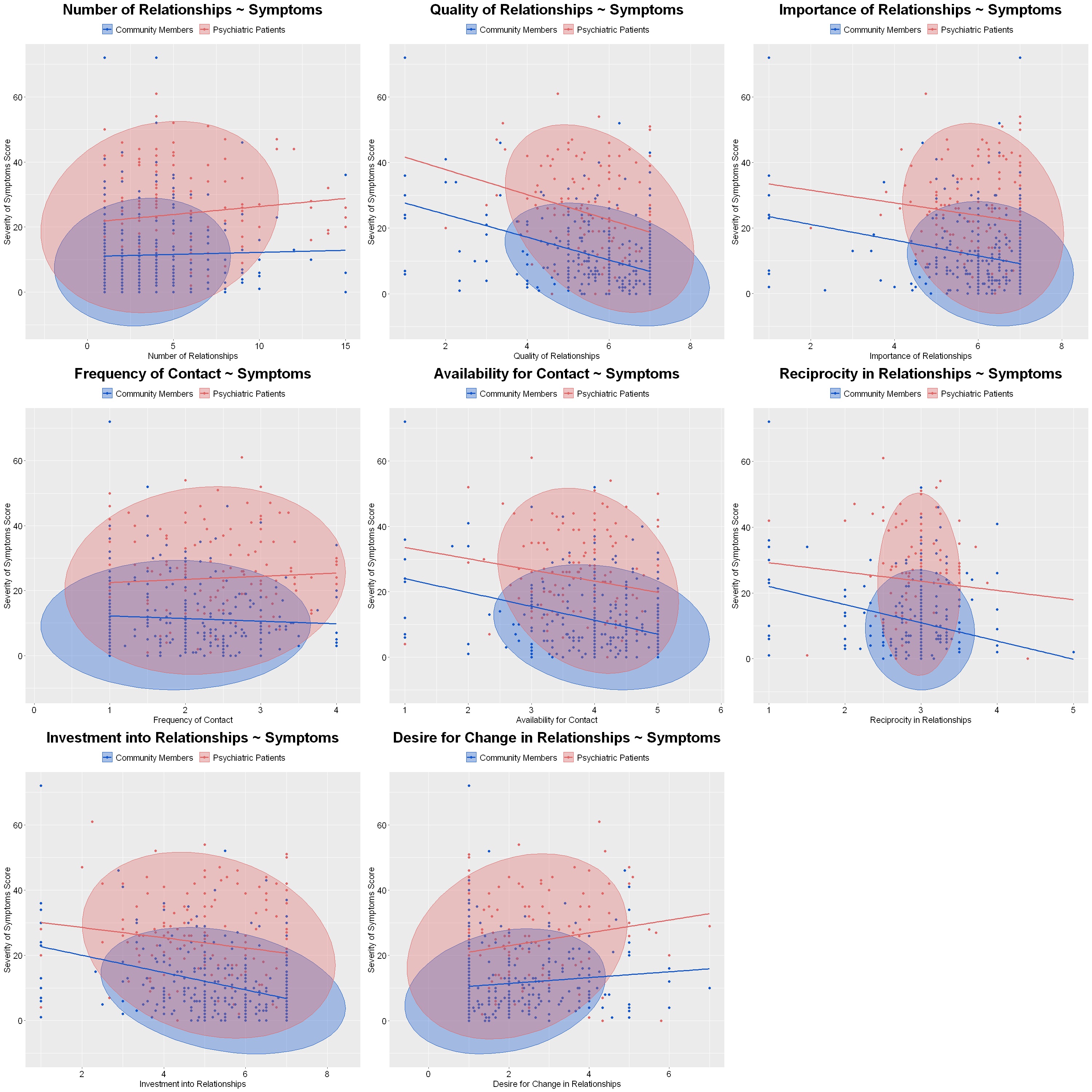

Supplement: Supplementary Figure B — Visualization of the association of the relationship attribute ratings and symptoms for both the community and the patient group. [file Image_2.JPEG]

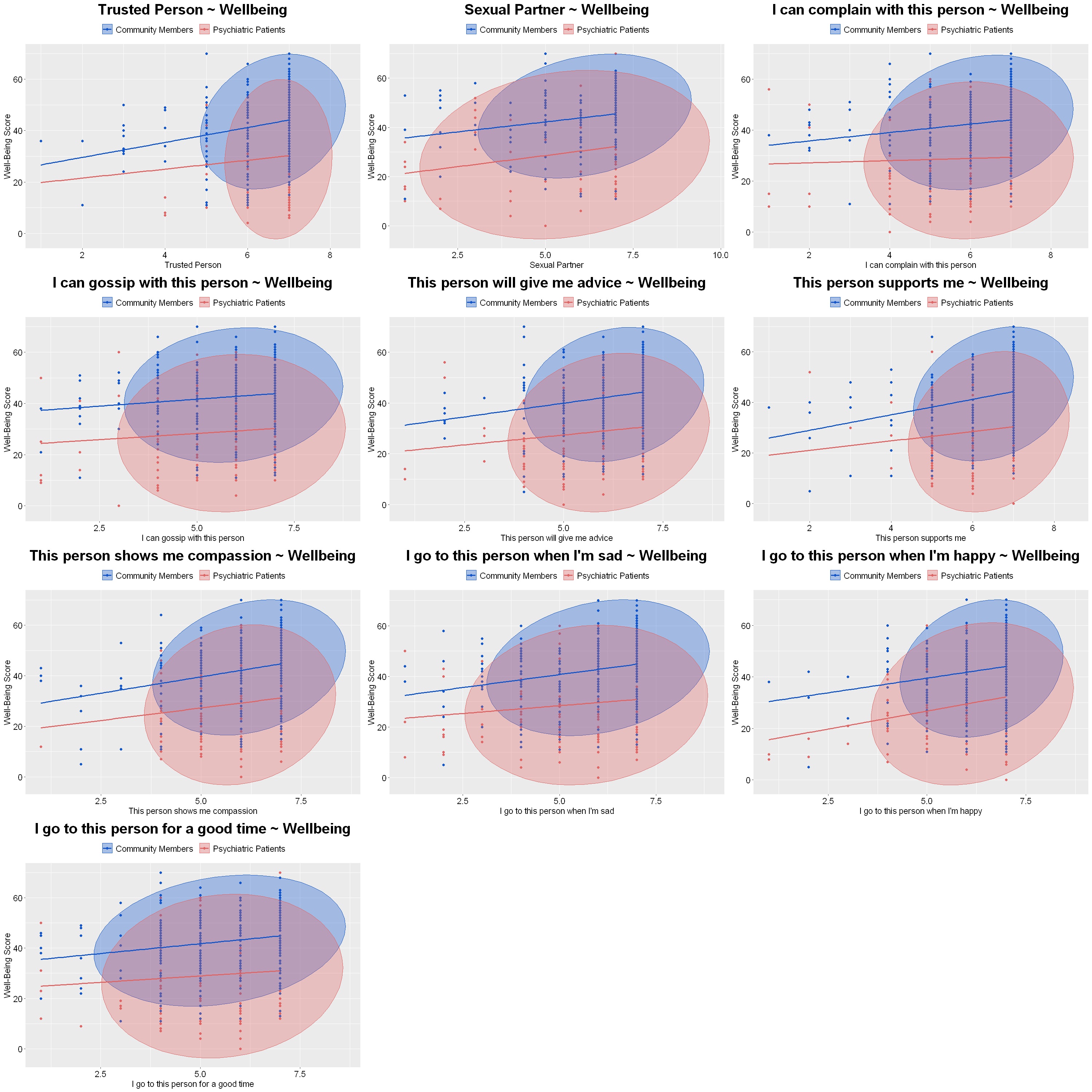

Supplement: Supplementary Figure C — Visualization of the association of the relationship function ratings and wellbeing for both the community and the patient group. [file Image_3.JPEG]

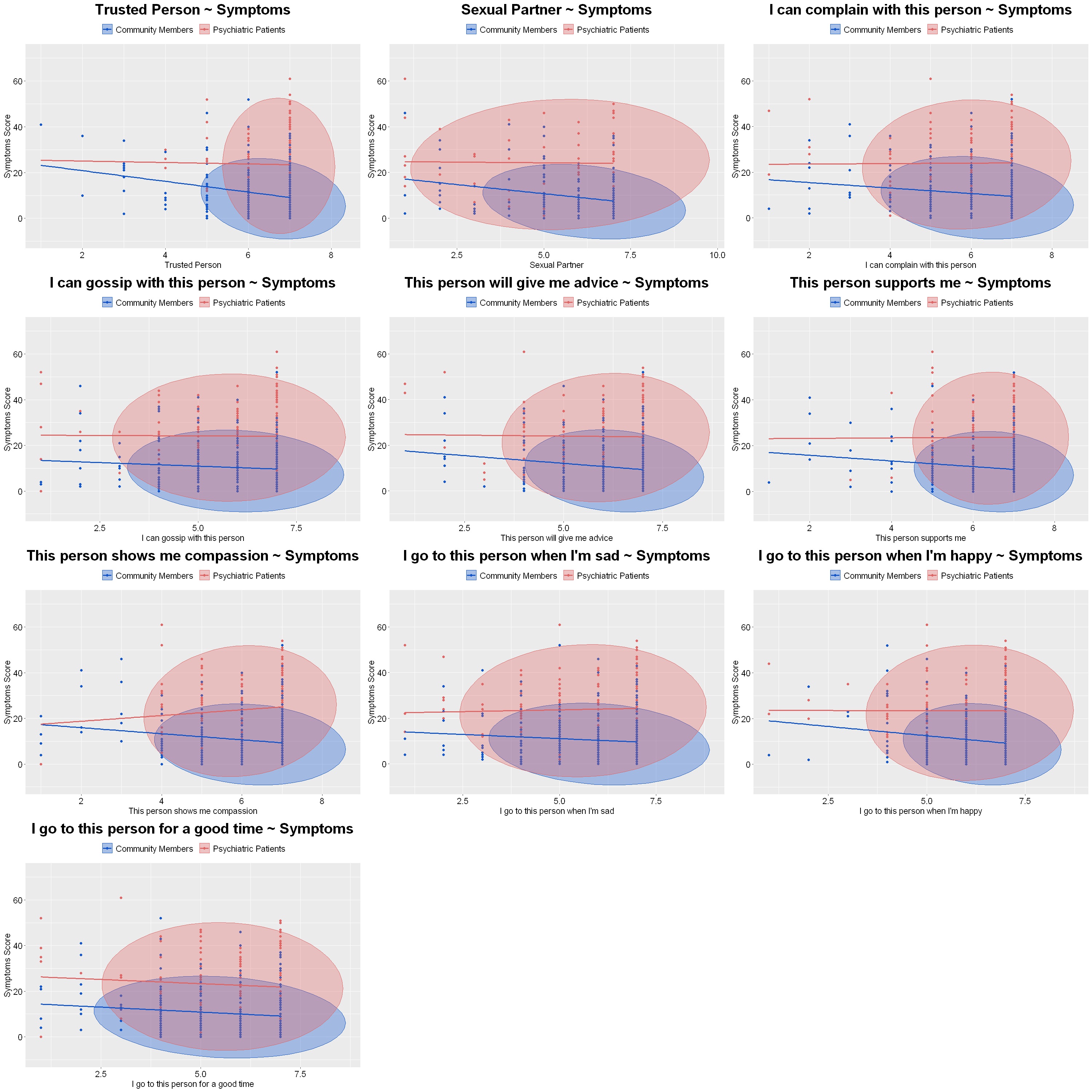

Supplement: Supplementary Figure D — Visualization of the association of the relationship function ratings and symptoms for both the community and the patient group. [file Image_4.JPEG]
